# Naloxegol and Postoperative Urinary Retention: A Randomized Trial

**DOI:** 10.3390/jcm11020454

**Published:** 2022-01-17

**Authors:** Alparslan Turan, Jonathan Fang, Wael Ali Sakr Esa, Hassan Hamadnalla, Steve Leung, Xuan Pu, Syed Raza, David Chelnick, Loran Mounir Soliman, John Seif, Kurt Ruetzler, Daniel I. Sessler

**Affiliations:** 1 Department of Outcomes Research, Cleveland Clinic, Cleveland, OH 44195, USA; jhanahou@gmail.com (J.F.); alisakw@ccf.org (W.A.S.E.); hhamadn1@hfhs.org (H.H.); steveleung57@gmail.com (S.L.); PuX@ccf.org (X.P.); syraza2016@gmail.com (S.R.); CHELNID2@ccf.org (D.C.); ruetzlk@ccf.org (K.R.); ds@or.org (D.I.S.); 2 Department of General Anesthesiology, Cleveland Clinic, Cleveland, OH 44195, USA; MOUNIRL@ccf.org (L.M.S.); SEIFJ@ccf.org (J.S.); 3 Department of Anesthesiology, Pain Management & Perioperative Medicine, Henry Ford Health System, Detroit, MI 48202, USA; 4 Department of Quantitative Health Sciences, Cleveland Clinic, Cleveland, OH 44195, USA

**Keywords:** anesthesia, urinary retention, naloxegol, residual bladder urine volume

## Abstract

Background: Naloxegol antagonizes peripheral opioid-related side effects without preventing opioid-related analgesia. However, the effect of naloxegol on opioid-induced bladder dysfunction remains unknown. Hypothesis: patients given naloxegol have lower residual bladder urine volume than those given placebo. Methods: 136 patients scheduled for elective hip and knee surgery were randomized to oral naloxegol or placebo given the morning of surgery, and on the first two postoperative mornings. Residual urine volume was measured ultrasonographically within 30 min after voiding once in the morning and once in the afternoon for two postoperative days. Opioid-related Symptom Distress Scale (ORSDS), the need for indwelling urinary catheterization, and quality of recovery (QoR) score were secondary outcomes. Results: 67 were randomized to naloxegol and 64 to placebo. We did not identify a significant effect on urine residual volume, with an estimated ratio of geometric means of 0.9 (0.3, 2.6), *p* = 0.84. There were no significant differences in ORSDS or QoR. There were 19 (29%) patients assigned to naloxegol who needed indwelling urination catheterization versus 7 (11%) patients in the placebo group, *p* = 0.012. Conclusions: Our results do not support use of naloxegol for postoperative urinary retention after hip and knee surgery.

## 1. Introduction

Post-operative urinary retention is a common postoperative complication, with an overall prevalence ranging from 2.1% to 70% depending on how the syndrome is defined and evaluated [1,2]. Post-operative urinary retention can cause long-term bladder dysfunction, chronic kidney disease, urinary tract infections, and even sepsis—consequently delaying hospital discharge and increasing hospital costs [1,2,3,4]. The risk of urinary retention is enhanced by certain types of surgery, advanced age, neuraxial blocks, and certain medications [1,2,3,4].

Post-operative opioids are an established cause of post-operative urinary retention [5,6]. Consistent with this mechanism, naloxone frequently ameliorates urinary retention [7]. However, naloxone also antagonizes analgesia which limits its clinical utility. Naloxegol is a naloxone analog with limited blood brain barrier permeability [8]. The drug therefore antagonizes peripheral opioid receptors and opioid-related side effects without preventing opioid-related analgesia which is centrally mediated [8]. The drug was approved by the US Food and Drug Administration in 2014 for opioid-induced constipation, [9] but whether naloxegol similarly reduces opioid-induced bladder dysfunction remains unknown.

The goal of this trial was to evaluate the effect of naloxegol on postoperative opioid-induced bladder dysfunction. Specifically, we tested the primary hypothesis that patients randomized to naloxegol (versus placebo) the morning of surgery and once each morning for each of the first two postoperative days have lower residual bladder urine volume. Secondarily, we compared naloxegol with placebo on opioid-related side effects, the need for indwelling urinary catheterization, and the quality of recovery.

## 2. Methods

Our investigator-initiated, double-blind, randomized trial was approved by the institutional review board of Cleveland Clinic. Consenting patients scheduled for elective hip and knee surgeries were enrolled with IRB approval (Number 17-765, 3 July 2017). The trial was registered at ClinicalTrials.gov (accessed on 9 November 2021) before the first patient was enrolled (NCT 03235739). There were no substantive changes to the protocol after initiation of patient enrollment. Our study was a priori defined as a pilot trial.

The trial enrolled non-pregnant adults scheduled for elective hip and knee surgery who were expected to have substantial postoperative pain requiring administration of opioids. Patients were excluded if they: (1) had hepatic disease (liver enzyme concentrations twice normal); (2) had kidney disease (serum creatinine > 2.0); (3) had history of bladder cancer; (4) were scheduled for perioperative nerve blocks; (5) took anticholinergic medications or had conditions or comorbidity causing urinary retention; (6) were expected to require an indwelling urinary catheter before or immediately after surgery due to immobility; (7) had pre-existing urinary tract infections or other urogenital comorbidity (incontinence, cysto-ureteric reflux, known bladder retention) or conditions which can cause urinary retention; (8) had severe peptic ulcer disease, diverticular disease, infiltrative gastrointestinal tract malignancies, or peritoneal metastases; (9) were known or suspected of having a disrupted blood brain barrier, including Alzheimer’s disease, stroke, poliomyelitis, cerebral palsy, multiple sclerosis, spinal lesions, and Parkinson’s disease; (10) had gastrointestinal obstruction or perforation; (11) were taking strong CYP3A4 inhibitors (some antibiotics, antifungals, protease inhibitors, and antidepressants) or strong CYP3A4 inducers, other opioid antagonists; or, (12) reported hypersensitivity to naloxegol or any of its metabolites.

### 2.1. Protocol

Patients were randomized 1:1 to oral naloxegol 25 mg (maximum allowed daily dose) or identical-appearing placebo, stratified on chronic opioid use. Study drug was provided by the research pharmacy. Therefore all investigators and clinicians were fully blinded. Chronic opioid use was defined as more than 30 consecutive days within three preoperative months, at a daily dose of 15 mg or more of morphine or equivalent. The trial drug was prepared by our research pharmacy; investigators, clinicians, and patients were thus fully blinded to treatment. It was given orally the morning of surgery, and on the first two postoperative mornings.

General anesthesia was induced and maintained per routine, usually with propofol and the volatile anesthetic sevoflurane. Intraoperative analgesia was restricted to short-acting opioids, usually fentanyl (boluses of 25–50 µg as needed). Spinal anesthesia was done with isobaric bupivacaine (10–15 mg). Indwelling urinary catheters were inserted in patients with an expected surgical duration exceeding 3 h, and in those in whom meticulous fluid status monitoring was desired. Intraoperative and postoperative fluid management was entirely at the discretion of the responsible clinicians.

In the post-anesthesia care unit and throughout the postoperative period, patients were managed at the discretion of the surgical team and the staff anesthesiologist. Intermittent urinary catheterization was used in postanesthesia care unit and/or surgical ward when medically indicated, generally when bladder volume was believed to exceed 400 mL. Patients were given intravenous boluses of fentanyl 25–50 µg or hydromorphone (0.2–0.4 mg) as needed at 10-min intervals in the post-anesthesia recovery unit to target pain scores at rest < 4 on a 0–10 verbal response scale.

Clinicians managing postoperative pain on surgical wards were blinded to aims of the study and adjusted opioid analgesia (intravenous boluses of hydromorphone (0.2–0.4 mg) or fentanyl (25–50 µg)) as necessary to target pain scores at rest <4 on a 0–10 verbal response scale throughout hospitalization. When patients were able to tolerate oral intake, they were transitioned to oral opioids (hydromorphone, oxycodone) per clinical routine.

### 2.2. Measurements

Demographic and baseline characteristics were retrieved from electronic medical records for patients. Trial data were stored in a custom Redcap database.

The primary outcome, residual urine volume, was measured within 30 min after voiding using a battery-powered, portable ultrasound bladder scan BVI 3000 (Verathon, Bothell, WA, USA). The device provides three-dimensional images of the bladder and uses automated technology to estimate bladder volume. Portable bladder ultrasound devices are accurate, reliable, cost-effective, and noninvasive. Bladder ultrasound estimates of residual volume are as accurate as catheterization with no meaningful effects of age, sex, or body mass index [10,11].

The ultrasonic bladder scanner was positioned on the suprapubic area and held stationary during measurement scanning. The diameter of the bladder and volume of urine was calculated from the scan data. Scans were repeated three times and the results averaged. Bladder scans were performed by trained physician investigators once in the morning and once in the afternoon on postoperative days 1 and 2, for a total of four measurements per patient. The surgical team was blinded to volume determined by scanning. Presence of indwelling bladder catheters was recorded.

Secondary outcomes included opioid-related side effects, the need for indwelling urinary catheterization, and quality of recovery (QoR) score. Opioid-related Symptom Distress Scale (ORSDS) is a 4 point-scale that evaluates three symptom distress dimensions (frequency, severity, bothersomeness) for opioid-related side effects [12]. The 12 elements of the ORSDS are nausea, vomiting, constipation, difficulty passing urine, difficulty concentrating, drowsiness, lightheaded, fatigue, feeling confused, itchiness, dry mouth, and headache [12]. The ORSDS questionnaire was administered by a trained investigator on first and second postoperative days while patients remained hospitalized.

Quality of recovery is a validated scoring system that quantifies patients’ early postoperative health status with range of 0–150 [13]. We used the 15-question version, the QoR-15, [13] on the second postoperative day or the day of discharge if earlier. The minimal clinically important difference was accepted as 8.0 [14].

There were also two a priori exploratory outcomes: (1) satisfaction with the quality of recovery, as measured on a subjective 100-point scale (where 0 means not satisfied at all and 100 means completely satisfied); and, (2) length of hospital stay after surgery.

### 2.3. Statistical Analysis

The statistical analysis plan was developed before patient enrollment and was included in our IRB application. Analyses were modified intent-to-treat and included all randomized patients who received some study drug. Demographic and baseline characteristics were summarized using appropriate statistics (i.e., means ± standard deviations, medians [Q1, Q3], or N (%)). Baseline variables with absolute standardized differences (ASD) > 0.34 (i.e., 1.96 × $\sqrt{\frac{1}{n_{1}}+\frac{1}{n_{2}}}$) were considered as imbalanced [15] and adjusted for in all primary and secondary analyses. We used an overall alpha of 0.05 for both primary and secondary analyses, with a significance criterion of 0.05 for the primary analysis and 0.017 for each secondary analysis (i.e., 0.05/3, Bonferroni correction).

For the primary analysis, urine residual was to be log-transformed if it did not meet normality assumption. We used a linear mixed effects model adjusting for within-subject correlation (using a first order autoregressive correlation structure) across the four time points, where the random effects were subject and fixed effects were time, treatment, and chronic opioid use. First, we tested the group-by-time interaction to assess whether the treatment effect differed over time (the significance level of interaction was 0.20). If it appeared to differ, we would report the treatment effect separately for each measurement time using a Bonferroni correction. Absent an interaction, we would assess the group difference on the volume of residual urine collapsing over time. We would also test if there was different treatment effect among chronic opioid users and nonusers by adding an interaction term with treatment.

We used multiple imputation for longitudinal data to impute missing values of the repeated measurements of the primary outcome. Fully conditional specification (FCS) method was used to impute missing values. Then the 10 imputed complete datasets were analyzed to obtain the pooled results. This imputation was completed using MI (multiple imputation) procedure in SAS. The results of analysis using complete cases are reported as a sensitivity analysis. Because the log-transformed primary outcome was not normally distributed, we did another sensitivity analysis using Wilcoxon rank sum test to compare groups over 4 time periods by pooling all the measurements in each group.

For the secondary analysis, we assessed the treatment effect on ORSDS by fitting a linear mixed model assuming an autoregressive correlation structure. The effect of naloxegol on the incidence of the need of indwelling urinary catheterization was assessed using Pearson chi-square test. The QoR score was compared between treatment and control group with a Wilcoxon rank sum test. Pain management satisfaction scores and duration of hospitalization were summarized for exploratory analysis.

### 2.4. Sample Size

Our sample size estimate was based on the linear mixed effects model for repeated measured data to test the primary hypothesis that patients who received Naloxegol 25 mg given once in the morning every day would have lower mean volume of residual urine in bladder when compared to placebo. Sixty-two patients per group were needed to have 90% power at a two-sided alpha level of 0.05 to detect a difference of 24 mL with an assumed standard deviation of 60, which is equivalent to a mean ratio of 0.80 if the mean of control group is assumed to be 125. To be conservative we assumed four measurements per patient with a correlation of 0.3. Assuming 10% loss to follow-up, we needed 12 more patients to reach the planned power with an end-sample size of 136. In addition to the 136 patients, we enrolled two pilot patients whose data were not considered for analysis to test feasibility of recruitment, protocol adherence, randomization process, and data collection. Data quality was confirmed at 33% enrollment.

## 3. Results

**Figure 1:**
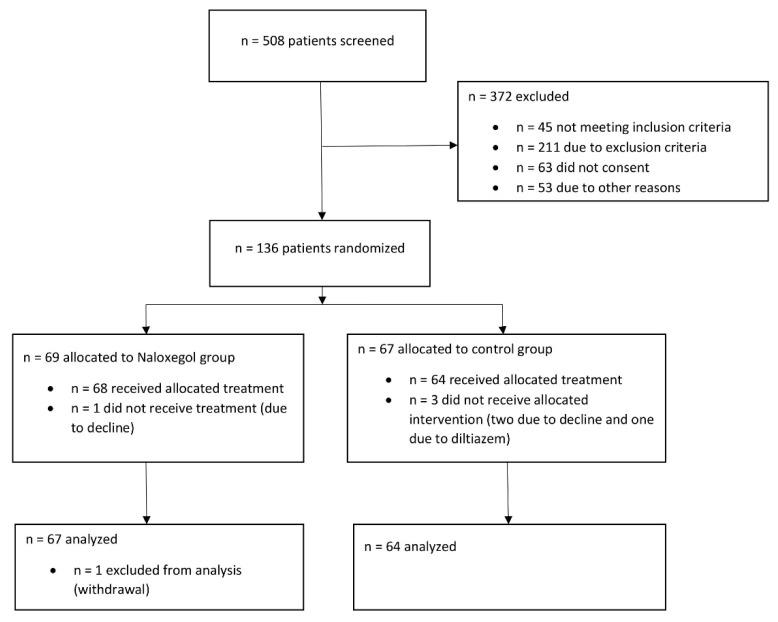
Flowchart of screening, randomization and withdrawal of patients.

A total of 136 patients were enrolled, although five patients withdrew and were excluded from the analysis (Figure 1). Among the remaining 131 patients, 67 were randomized to naloxegol and 64 to placebo. Baseline and demographic characteristics are summarized in Table 1. All patients had hip and knee surgery. There were some imbalances observed in baseline characteristics. But since our trial was randomized, imbalance should reflect chance imbalances rather than confounding; therefore, no adjustment was made during primary and secondary analyses.

Post-operative urinary retention independent of group allocation was (median (Q1, Q3)) 102 (7, 209) mL in the morning of postoperative day 1, 34(0, 206) mL in the evening of postoperative day 1, 29 (0, 144) mL the morning of postoperative day 2, and 13 (0, 76) mL the evening of postoperative day 2.

### 3.1. Primary Analyses

The median (Q1, Q3) of urine residual volume was 62 (5, 187) in patients given naloxegol and 114 (13, 247) in those given placebo from day 1 morning to 0 (0, 122) (treatment) and 28 (0, 66) (control) at day 2 afternoon. After log transformation, urine residual volumes were more normally distributed. The treatment-by-time interaction was not significant (*p* = 0.77). We did not find significant treatment effect on urine residual volume, with an estimated ratio of geometric means of 0.90 (0.32, 2.55), *p* = 0.84.

**Figure 2:**
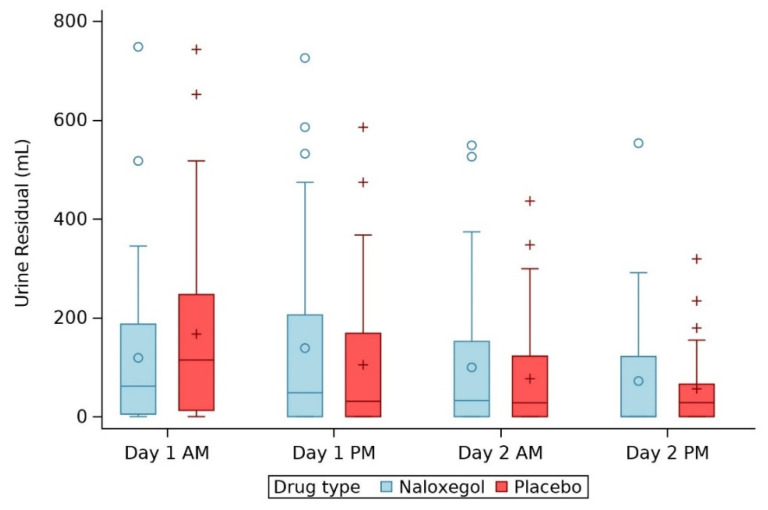
Boxplot of urine residual by time. The line in the box represents the median of each group. The blue circle in the box represents the mean of urine residual in naloxegol group and blue circles outside the box represent the outliers. The red “+” sign in the box represent the mean of urine residual in placebo group and red “+” sign outside of box represent outliers.

**Figure 3:**
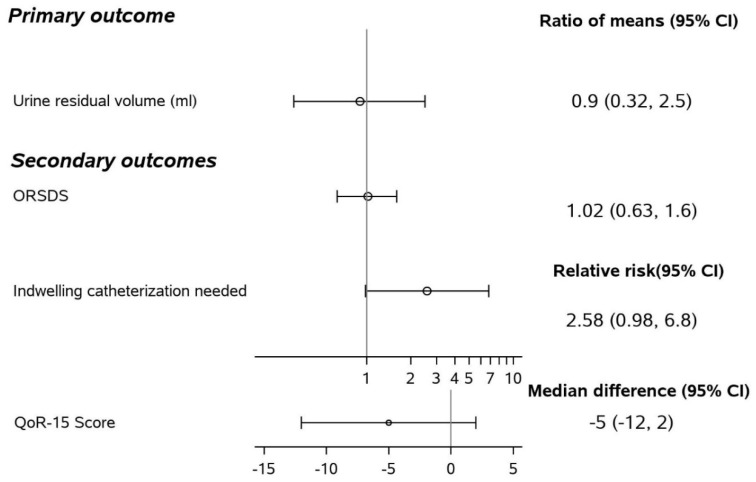
Forest plot of primary and secondary outcome, Naloxegol versus placebo. ORDS: Opioid–Related Symptom Distress Scale. QoR-15 score: Development and Psychometric Evaluation of a Postoperative Quality of Recovery Score.

The treatment effect did not differ between chronic opioid users and non-users (interaction *p* = 0.92). According to the complete case analysis, naloxegol did not significantly affect the mean urine residual volume compared to placebo over postoperative day 1–2, with estimated ratio of geometric means of 0.82 (0.26, 2.56), *p* = 0.73 (Table 2 and Figure 2 and Figure 3). The treatment effect also did not differ across time or across chronic opioid user groups in the complete case analysis.

The sensitivity analysis using Wilcoxon Rank Sum test to compare groups on the patients over those four time periods did not show significant treatment effect on the urine residual volume with an estimated median difference of −3 mL (−7, 1, *p* = 0.63).

In 17 (13%) patients, all four measurements on primary outcome were missing mostly because of indwelling bladder catheters. The fraction of patients with missing values was similar in the naloxegol and placebo groups.

### 3.2. Secondary Analyses

The median (Q1, Q3) of opioid-related side effects (ORSDS) score at postoperative day 2 was 0.34 (0.2, 0.64) for treatment and 0.34 (0.13, 0.62) for control (Table 3, Supplementary Material Tables S1 and S2). There were 19 (29%) patients assigned to naloxegol who needed indwelling urination catheterization versus seven (11%) patients in the placebo group. Treated patients had borderline higher risk of needing an indwelling catheterization, with an estimated relative risk (98.3% CI) of 2.6 (0.98, 6.8), *p* = 0.012 from Pearson chi-square (Table 3, Supplementary Material Tables S1 and S2) (*p* = 0.022 from continuity adjusted chi-square). There were also no significant differences in opioid-related side effects or quality of recovery.

### 3.3. Exploratory Analyses

The median (Q1, Q3) of pain management satisfaction at discharge was 90 (80, 100) for patients given naloxegol and 90 (83, 100) for patients given placebo. Patients given naloxegol stayed in the hospital for a median (Q1, Q3) of 3.2 (2.3, 4.3) days, and those assigned to placebo stayed 2.4 (2.2, 3.3) days. The median (Q1, Q3) of postoperative opioid use in morphine equivalent was 45 (25, 83) mg for the naloxegol group and 43 (23, 81) mg for the placebo group.

## 4. Discussion

Current care approaches including early mobilization and goal-directed fluid management, may reduce postoperative urinary retention. On the other hand, urinary retention is promoted by spinal anesthesia, advanced age, male sex, large intraoperative fluid volumes and opioids [2,3,4,16]. We found that urinary retention was common after hip and knee surgery and that residual urine volumes were high, especially on the first operative day. It is possible that high volumes are consequent to enhanced recovery protocols which discourage intraoperative bladder catheterization, and promote removing necessary catheters at the end of surgery.

Naloxegol did not significantly reduce mean urine residual volume. However, our pilot trial had limited power; consequently, confidence intervals around the difference were wide, ranging from a 74% reduction to a 2.4-fold increase in geometric means. Thus, while our results are not encouraging, we cannot conclude with any certainty that naloxegol has no clinically meaningful benefit on urinary retention. We used the highest dose of naloxegol approved for opioid-induced bowel dysfunction [17]. It remains possible that higher doses are needed to prevent urinary retention. Interestingly, another peripheral opioid antagonist methylnaltrexone was effective in nonsurgical volunteers in reversal of urinary retention supporting the theory that peripheral mechanisms play a role in opioid-induced urinary retention [18]. 

Curiously, patients randomized to naloxegol were about twice as likely to require indwelling postoperative bladder catheters. However, the number of events was small and our trial was not even remotely powered for this dichotomous outcome. Given the lack of a causative mechanism, the observation is probably spurious.

Most opioid-related side effects are centrally mediated. For example, 9 of the 12 consequences evaluated by the ORSDS are completely or mostly centrally mediated. It is therefore unsurprising that naloxegol—which has limited central activity—had no apparent effect on ORSDS scores. Quality of recovery would be impaired by ileus, but constipation, while common after major abdominal surgery, is relatively rare in patients who have hip surgery. It is therefore unsurprising that naloxegol did not reduce opioid-related side effects or quality of recovery. Since naloxegol is peripherally restricted, the drug does not prevent opioid-induced analgesia [8]. As might therefore be expected, opioid use and pain scores were similar in each group.

The most obvious limitation of our trial is restricted power even for our primary outcome due to a larger observed standard deviation for the primary outcome (SD = 150) than used in sample size estimate (SD = 60), and fewer secondary outcomes than expected. Only 15% of randomized patients required postoperative bladder catheterization which further reduced power. An additional limitation is that our protocol was pragmatic and did not restrict intraoperative fluids, choice of general or spinal anesthesia, or use of other drugs, some of which could have contributed to postoperative urinary retention. Our pilot trial was nonetheless successful in providing good estimates of the variance and incidence of our outcomes that will guide future trials.

## 5. Conclusions

Residual urine volumes were similar in patients randomized to 25 mg oral naloxegol or placebo over the initial 2 days after hip and knee surgery, although confidence intervals were wide. It thus seems likely that postoperative urinary retention is largely driven by factors other than postoperative opioids. Furthermore opioid-related side effects and quality of recovery were similar in the naloxegol and placebo groups. Our results, although statistically weak, do not support use of naloxegol for postoperative urinary retention after hip and knee surgery.

## Figures and Tables

**Table 1:** Baseline characteristics by group.

Factor	Naloxegol (*n* = 67)	Placebo (*n* = 64)	ASD
**Demographic**			
Age (yrs.)	64 ± 13	62 ± 11	0.177
Female	32 (48)	32 (50)	0.046
Race ^3^			0.180
Caucasian	59 (89)	57 (92)	
African American	6 (9)	5 (8)	
Other	1 (2)	0 (0)	
BMI (kg/m^2^)	29 ± 6	30 ± 7	0.059
ASA status			0.374
1	1 (2)	1 (2)	
2	8 (12)	15 (23)	
3	52 (77)	46 (72)	
4	6 (9)	2 (3)	
Apfel PONV score	2.0 (2.0, 3.0)	2.0 (2.0, 3.0) ^1^	0.138
Chronic opioids use	36 (13)	32 (13)	0.03
Surgery type			0
Hip	67 (100)	64 (100)	
Knee	0 (0)	0 (0)	
Surgery duration, hour	3.6 (2.9, 4.7)	3.5 (2.9, 4.5) ^1^	0.062
**Medical History**			
Kidney Disease	4 (6)	1 (2)	0.231
Chronic Pulmonary Disease	9 (13)	9 (14)	0.018
Obstructive Sleep Apnea	9 (13)	6 (10)	0.123
Diabetes Mellitus	7 (10)	7 (11)	0.021
Myocardial Infarction	6 (9)	4 (6)	0.098
Ischemic Heart Disease	14 (21)	10 (16)	0.130
Neurologic Diseases	7 (10)	12 (19)	0.244
Chronic Pain	1 (2)	5 (8)	0.308
Current Smoker	9 (13)	7 (11)	0.071
Drug User	2 (3)	5 (8)	0.219
Alcohol Abuse	10 (15)	7 (11)	0.254
Cancer	10 (15)	6 (10)	0.165
Anesthesia type ^1^			0.21
General anesthesia	46 (69)	45 (71)	
Spinal anesthesia	21 (31)	18 (29)	
**Intraoperative variables**			
Colloids, ml	0 (0, 500)	0 (0, 250) ^1^	0.101
Crystalloids, ml	2000 (1400, 2700)	1800 (1400, 2700) ^1^	0.051
RBC, cc	0 (0, 0)	0 (0, 0) ^1^	0.336
Platelets, cc	0 (0, 0)	0 (0, 0) ^1^	0.173
Urine, cc	0 (0, 420)	0 (0, 460) ^1^	0.081
Intraoperative opioid use	20 (5.0, 30.3)	20 (7.5, 25.3) ^1^	0.01

BMI: body mass index; ASA: American Society of Anesthesiologists; PONV: Postoperative nausea and vomiting. Statistics presented as means ± SDs, medians (Q1, Q3), or N (column %). ASD: absolute standard difference; standardized difference is the difference in means or proportions divided by the pooled standard deviation. ASD larger than 0.34 was considered as imbalanced. Superscripts of summary statistics represent missing number.

**Table 2:** Treatment effect on urine residual.

	Postoperative Day	Total (*n* = 114) ^b^	Naloxegol (*n* = 59) ^a,b^	Placebo (*n* = 55) ^a,b^	Ratio of Geometric Means (95% CI) ^c^	*p*-Value
Urine residual volume (mL)	Overall				0.90 (0.32, 2.55)	0.84
	Day 1 AM	102 (7, 209)	62 (5, 187)	114 (13, 247)		
	Day 1 PM	34 (0, 206)	48 (0, 206)	31 (0, 169)		
	Day 2 AM	29 (0, 144)	32 (0, 152)	28 (0, 122)		
	Day 2 PM	13 (0, 76)	0 (0, 122)	28 (0, 66)		

CI: confidence interval. ^a^: The primary analysis included 114 patients due to complete missing of 17 patients (nine in control group and eight in treatment group). ^b^: Summary statistics of primary outcome urine residual was reported as median (Q1, Q3) during the first two postoperative days. ^c^: The ratio of geometric means (Naloxegol versus placebo) was estimated using a linear mixed-effect model with repeated measures assuming a first order auto-regressive correlation structure (known as AR(1)) after multiple imputation for missing data. The random effect was subject and the fixed effects included time, treatment, and chronic opioid use. The treatment effect did not differ across time (*p* = 0.77) or across chronic opioid use (*p* = 0.92).

**Table 3:** Treatment effect on secondary outcomes.

	Postoperative Day	N Missing	Naloxegol (*n* = 67)	Placebo (*n* = 64)	Effect Estimate (98.3% CI)	*p*-Value ^a^
					Ratio of geometric means	
ORSDS ^b^	Overall				1.02 (0.63, 1.67)	0.91
	Day 1	3	0.53 (0.34, 0.79)	0.45 (0.31, 0.81)		
	Day 2	24	0.34 (0.2, 0.64)	0.34 (0.13, 0.62)		
					Relative risk	
Indwelling need ^b^	Until discharge	4	19 (0.3)	7 (0.1)	2.58 (0.98, 6.80)	0.012
					Median difference	
QoR-15 Score ^b^	Discharge day	9	119 (103, 131)	123 (115, 133)	−5 (−12, 2)	0.08

ORSDS: opioid-related side effects; QoR: quality of recovery. ORSDS is a 4-point scale score, where the higher represents more opioid-related side effects. Qor15 score ranges from 0-150, where the higher score represents better recovery. ^a^: Bonferroni correction was made due to multiple testing (*p* = 0.017 i.e., *p* = 0.05/3). ^b^: The treatment effect on ORSDS was estimated using a mixed effects model assuming auto-regressive correlation structure and the outcome was log-transformed. Pearson chi-square test was used to test the effect on the indwelling urine catheterization use. Hodges–Lehmann estimation was used on QoR-15 scores due to non-normality.

## Data Availability

The data are available collaboratively by contacting the corresponding author.

## Supplementary Materials

The following supporting information can be downloaded at: https://www.mdpi.com/article/10.3390/jcm11020454/s1. Table S1: Summary of incidence of each opioid-related side effect item by group, Table S2: Summary of functionality.
