# Supplementary material for: Naloxegol and Postoperative Urinary Retention: A Randomized Trial
Source: J Clin Med. 2022 Jan 17;11(2):454. doi: 10.3390/jcm11020454 (PMC8780376; doi:10.3390/jcm11020454)
Supplement: Supplementary file 1 [file jcm-11-00454-s001.zip › jcm-1481275-supplementary.pdf]

Table S1. Summary of incidence of each opioid-related side effect item by group

Side effect	Naloxegol (*N* = 67)	Placebo (*N* = 64)
Nausea	27 (40)	24 (39)
Vomiting	7 (10)	7 (11)
Constipation	12 (18)	14 (23)
Difficulty passing urine	17 (26)	9 (15)
Difficulty concentration	18 (27)	15 (25)
Drowsiness	35 (53)	30 (49)
Feeling dizzy	26 (39)	15 (25)
Feeling confused	6 (9)	6 (10)
Fatigue	38 (58)	29 (48)
Itchiness	12 (18)	18 (30)
Dry mouth	57 (86)	52 (85)
Headache	18 (27)	10 (16)

The data was summarized as N (%). The incidence of each item was considered as positive if the patient had this symptom at any postoperative day 1 or day 2.

Table S2. Summary of functionality

**Functionality**	**N missing**	**Naloxegol (*N* = 67)**	**Placebo (*N* = 64)**
Bathing	4	32 (49)	39 (63)
Toileting	4	49 (75)	50 (81)
Walking	4	55 (85)	54 (87)
Ileus	4	1 (2)	3 (5)
Flatus	4	59 (91)	60 (97)
First bowel movement	16	12 (20)	6 (11)

Data was summarized as N (%). The incidence of each item was considered as positive if the patients had it present at any postoperative day 1 or day 2.
